# 

**Published:** 1939

**Authors:** 


### CONTENTS.

| | Page |
|---|---|
| Medicine and the Drama. By Leonard A. Moore, M.B., Ch.B. .. | 1 |
| The Management of some Obstetric Emergencies. By Gilbert I. Strachan, M.D., F.R.C.P., F.R.C.S., F.R.C.O.G. .. .. .. | 25 |
| Recent Advances in Surgery. By W. H. Ogilvie, M.D., F.R.C.S. | 37 |
| Reviews of Books .. .. .. .. .. .. .. | 63 |
| Obituaries: J. E. Shaw, M.B., C.M. Edin. .. .. .. .. | 70 |
| Conrad Fitzgerald, M.R.C.S., L.S.A. .. .. .. | 71 |
| The Medical Library of the University of Bristol .. .. | 72 |
| Publications Received .. .. .. .. .. .. .. | 74 |
| Local Medical Notes .. .. .. .. .. .. .. | 76 |

